# Severe Respiratory and Hemodynamic Failures following Successful Spontaneous Pneumothorax Drainage

**DOI:** 10.1155/2021/6677656

**Published:** 2021-05-11

**Authors:** Nicolas Mayeur, Samuel Groyer, Sylvie Vimeux, Jérôme Roustan

**Affiliations:** ^1^Cardiac and Thoracic Surgery, Clinique Pasteur, Toulouse, France; ^2^Polyvalent Intensive Care Unit, CH Montauban, Montauban, France

## Abstract

Spontaneous pneumothorax is a frequent situation in respiratory medicine, and its treatment is based on conservative treatment or pleural drainage. Reexpansion pulmonary edema (REPE) is often a mild complication following pneumothorax drainage. We report here a severe case of unilateral REPE following spontaneous pneumothorax drainage associated with major pulmonary plasmatic leakage. The clinical presentation was severe and sudden with respiratory and circulatory failures. Initial resuscitation was mostly based on prone and head-down positioning in association to fluid expansion and mechanical ventilation. On the basis of this clinical case report, we strongly suggest to think about severe pulmonary reexpansion edema when respiratory and hemodynamic failures occur few hours after pneumothorax-related efficient pleural tube drainage.

## 1. Introduction

Spontaneous pneumothorax treatment is a common pathology in respiratory medicine. Severe reexpansion pulmonary edema (REPE) is a rare complication of pneumothorax treatment. The incidence of mild REPE seems to vary between 0.9% and nearly 75% of patients treated for spontaneous pneumothorax [1] This variation is mostly a consequence of various diagnostic criteria or radiologic procedures. Nevertheless, most clinical presentations of REPE are mild, and treatment is mostly symptomatic even if some authors have used steroids [2].

## 2. Case Presentation

**Figure 1:**
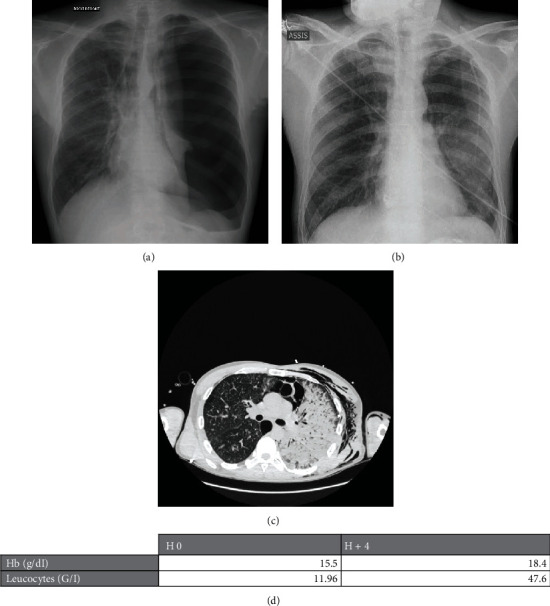
Complete left-sided pneumothorax (a); complete pulmonary reexpansion just after drainage (b); reexpansion pulmonary edema 4 hours after drainage (c); hematological variations before drainage and 5 hours later just after reexpansion pulmonary edema onset (d).

We report here the case of a severe REPE. A 40-year-old man was admitted in the emergency ward for chest pain and dyspnea that started 7 days before without any traumatic history. The patient was a former intravenous and inhaled toxicomania and an active smoker. He suffered from Verneuil's disease. His vital signs after admission were a tachypnea at 25/min, sinus tachycardia at 110/min, normal arterial pressure, and a low blood oxygen saturation at 92% without oxygen. Clinical examination and an inspiratory chest X-ray showed a complete left-sided pneumothorax with a right-sided deviation of the heart outline (Figure 1(a)). After a 500 ml crystalloid fluid expansion, oxygen therapy, and analgesia, clinical examination was unchanged, and an intercostal tube drainage (8-Fr Pleurocath®, Plastimed, Le Plessis Bouchard, France) was inserted in the left second antero-lateral intercostal space in order to treat this clinically unstable patient with large pneumothorax. A 20 cm H20 suction was applied after few minutes of drainage as recommended in our institution. Both X ray and pleural echography following drainage showed a fully reexpanded left lung but with an abnormal parenchyma (Figure 1(b)). This insertion resulted in a decrease in tachycardia (HR = 90/min), in dyspnea (respiratory frequency at 20/min) and an improve in patient comfort. Oxygen saturation was 100% with 2 liters/minute oxygen. 3 hours and 30 minutes after drainage, he presented a sudden and severe dyspnea with asphyxia: agitation, blood oxygen saturation at 70% despite 15 liters oxygen, 170 bpm sinus tachycardia, and subcutaneous emphysema. Chest echography revealed both left-sided basal pulmonary condensation and loss of apical lung sliding. PaO2 was less of 50 mmHg (6.66 kPa) despite of 15 liters oxygen. Systolic arterial pressure was 80 mmHg with cutaneous sign of shock. A second chest drainage was performed in emergency with a small improve in his clinical status. Analgesia, anxiolytic, and noninvasive ventilation were performed without success and persistent distress ordered to perform invasive mechanical ventilation. After anesthesia, endotracheal tube insertion revealed abundant foamy sputum. Shock became severer (systolic arterial pressure 70 mmHg) and 1000 ml of crystalloid fluid challenge and noradrenalin were introduced before biological tests. In order to exclude iatrogenic vascular traumatism, a chest radiography and a CT scan were quickly performed. CT scan revealed an unknown bilateral centrolobular and paraseptal emphysema, an incomplete pulmonary reexpansion with persistent pneumothorax, a pneumomediastinum, and a massive left-sided pulmonary edema with alveolar condensation (Figure 1(c)). Biological results obtained just after CT scan return were remarkable (Figure 1(d)) when compared to the emergency department entry's results 5 hours before. Arterial blood gas analysis revealed a profound hypoxemia with a 68 PaO2/FiO2 ratio and a respiratory acidemia (pH 7.17; PaCO2 75 mmHg). Echocardiography showed both low right and left filling pressures and hyperkinetic ventricles. We decided to conclude to a severe REPE. Lateral positioning failed to improve hematosis and prone positioning associated with a transient head down in order to improve sputum aspiration, and cardiac loading was performed with success. Massive crystalloid fluid expansion (3500 ml) improved arterial pressure in association with norepinephrine. Numerous bacteriological samples including hemocultures and tracheal aspirations remained sterile. Hemodynamic definitively improved after 6 hours of fluid infusion and continuous hemodynamic monitoring. Respiratory failure with hypoxemia and slight hypercapnia under protective ventilation remained challenging during 4 days though extracorporeal membrane oxygenation was not necessary. Orotracheal extubating was performed at day 7. His pulmonary evolution was marked by a persistent gas leakage. Drainage was successfully stopped at day 20, and hospital discharge occurred 1 day later.

## 3. Discussion

We report here for the first time a severe REPE complicated by both circulatory and respiratory failures and successfully treated by transient both prone and head-down positioning in association to other resuscitation strategies. Although exact etiologies and mechanisms are still not fully understood, some pathogenic factors for REPE have been suggested such as an increase in vascular permeability through influx of inflammatory cells and free radical accumulation in association to a sudden increase in local perfusion following both lung reexpansion and reversal of hypoxic vasoconstriction [3]. Numerous risk factors have been postulated to contribute to REPE [4]. In this patient, the chronicity and completeness of lung collapse and the application of negative suction are both risk factors previously described in various studies. Increased pulmonary vascular permeability, decreased surfactant activity, pulmonary artery pressure change, or diabetes have also been suggested to increase the risk of REPE [3]. The delay between drainage and REPE and the CT scanner findings were comparable in this patient, although severer for the latter, as in previously described REPE [5] 5/4/2021 3:39:00 AM. This patient suffered from Verneuil's disease that can be associated with other comorbidities such as chronic lung disease [6]. Nevertheless, as far as we know, no causal relationship has been showed between this pathology and sudden lung edema or pneumothorax [7]. Intravenous and inhaled toxicomania could cause both pneumothorax and acute lung edema [8]. The patient's drug consumption was stopped for more than one year yet, and if such background could have increased the risk of pneumothorax, we do suggest that it could not stand in this case as a relevant differential diagnosis from REPE.

In the medical case reported here, we described a REPE which is a well-known complication following pneumothorax treatment. However, the suddenness and intensity of both respiratory distress and cardiovascular collapse are remarkable. Indeed, in association to the severer presentation (asphyxia), some biological abnormalities are noteworthy. On the first hand, the almost 3 g/dl sudden increase in hemoglobin concentration despite 1000 ml of crystalloid fluid expansion reflected the massive pulmonary extravascular plasmatic leakage. On the second hand, the 36 G/l increase in leucocyte reflected the systemic impact of this pulmonary edema. In order to explain such severity, the application of negative suction, although delayed, has possibly increased the intensity of REPE and should probably not be realized in a 7-day-old pneumothorax. Nevertheless, to our knowledge, avoiding suction has not been proven as being a protective factor for REPE. In this case, we report the treatment of this severe REPE by both prone and head-down positioning after the control of pleural drainage. This strategy was decided in order to improve both cardiac and respiratory functions. On the first hand, Trendelenburg positioning improves cardiac loading [9]. On the second hand, prone positioning is well documented as a resuscitation strategy during respiratory failure in order to improve ventilation/perfusion ratio or sputum drainage [10]. Trendelenburg positioning should be transient because of the risk of pulmonary aspiration.

## 4. Conclusion

Occurrence of respiratory and hemodynamic failures few hours after efficient pleural tube drainage is not a common situation. Physicians should in priority exclude not only compressive pneumothorax related to incomplete drainage, tube mispositioning, or clogged drain but also iatrogenic hemothorax related to cardiac or vascular injury. On the basis of this clinical case report, we strongly suggest to think about severe pulmonary reexpansion edema. Moreover, in case of both severe plasmatic leakage and foamy sputum, transient prone and Trendelenburg positioning should be discussed.

## Data Availability

Biological and radiographic data are available.

## Abbreviations

REPE: Reexpansion pulmonary edema.
